# Reductionism in Engram Neuroscience

**DOI:** 10.1111/ejn.70497

**Published:** 2026-04-20

**Authors:** Caitlin Mace, Fionn O'Sullivan, Scott R. Wilson

**Affiliations:** ^1^ University of Pittsburgh Pittsburgh Pennsylvania USA; ^2^ Trinity College Dublin Dublin Ireland; ^3^ University of Cambridge Cambridge UK

**Keywords:** explanation, memory engrams, methodology, reductionism

## Abstract

Engrams are a hypothetical construct in neuroscience used to explain memory phenomena. The search for engrams has been energised by the advent of certain reductionist methods that intervene at lower levels of organisation. We defend methodological reductionism as an essential research strategy but argue that such methods alone are insufficient for a complete explanation of memory. Relying solely on these methods perpetuates a field that is data‐rich but understanding‐poor. Explanations in memory neuroscience require theorising about engrams and the conditions for their discovery. This can be facilitated by an integrative pluralism, by which multiple competing models of causal processes are integrated within a broader theoretical framework. We propose that conceiving of engrams as multiply‐realisable, causal motifs—not the standard conceit of a stable, physical entity—is essential for a complete explanation of memory phenomena. As such, methodological reductionism and explanatory integrative pluralism are important conceptual tools in the neuroscientist's toolkit.

## Graphical abstract

Reductionist methods inspire several seemingly incompatible explanations and ontologies. But such adversarial approaches can be fruitful for theorising in neuroscience. We argue that reductionist methods are at their most useful when coupled with integrative practices that include multiple heterarchical levels of organisation, various kinds of analysis, competitive theory building and non‐reductionist research programmes.

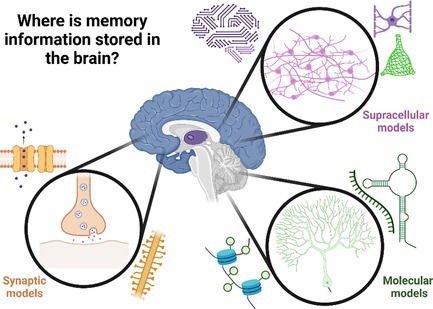

**Abbreviations**
CA1: cornu ammonis 1
EEG: electroencephalography
fMRI: functional magnetic resonance imaging
LTD: long‐term depression
LTP: long‐term potentiation
RNA: ribonucleic acid
STC: synaptic tagging and capture

## 1 Introduction

Neuroscience is a highly interdisciplinary field, a place where various scientific traditions intersect by virtue of the wish to understand nervous systems and behaviour. Walking down the aisles of a neuroscience conference can be an overwhelming experience. Given the sheer number of radically different research areas, it often appears that neuroscientists do not even speak a shared language. In addition to this, making progress today on a particular research question, even within a specific neuroscientific sub‐discipline, demands proficiency in a huge variety of experimental and computational tools, as well as the ability to interpret increasingly dense data objects generated by these methods. This trend towards gathering more data typically comes at the expense of theory, where hypotheses and ‘storytelling’ are derived post hoc from experimentation, in contrast to a theory‐driven scientific method (Platt 1964). The proliferation of reductionist methods has facilitated such a data‐first style of thinking and has even led to suggestions for a moratorium on experiments until theory is brought into balance.

The volume of results in neuroscience that now must be accounted for makes any attempt at *integrating* findings across this rich scientific landscape all the more paramount and challenging. We see risk in failing to promote and attempt integration in neuroscience, especially in the broader context of the increasing popularity of certain data‐science methods in the sciences generally. Echoing the warning most recently articulated by Messeri and Crockett, although data‐first approaches will lead us to generate even more research—and in yet more detail—we may resultantly understand less (Messeri and Crockett 2024). Integrating findings for fruitful theorising is moreover complicated by the diversity of findings at various levels of organisation. Some circumvent such integration by advocating a singular focus on lower levels of organisation. An upshot of our project is a reconciliation of reductionism with competing models developed from investigation into higher levels of organisation. Indeed, we think such integration will be fruitful for neuroscientific theorising.

We focus on different reductionist theses that feature prominently within neuroscience, namely, those that privilege the study of molecular and cellular processes and seek to explain phenomena solely in these terms (Bickle 2003). Higher‐level mechanisms, on such accounts, are considered to be merely ‘epiphenomena’—processes that we observe, but that play no causal role in cognition or behaviour.^1^ Alternatively, anti‐reductionist positions—such as those advocating population‐ or systems‐level research and explanations—hold that there are causally‐relevant properties at the population or circuit level that cannot be accounted for in the activity of individual neurons (Barack and Krakauer 2021; Ebitz and Hayden 2021). That new properties emerge at different scales in complex systems precludes accounting for everything at a fundamental level (Anderson 1972). At the very least, systems‐level research is needed to account for how the lower‐level parts are organised into mechanisms (Bechtel 2008). We aim to advance these debates by offering a middle‐ground position based on a distinction between methodological and explanatory reductionism.

The search for memory engrams—the physical basis of memory storage—offers a compelling case study for the role and limits of scientific reductionism. This paper evaluates the search by focusing on the reduction of biological memory mechanisms in practice. We therefore concentrate on molecular and cellular interventions, such as optogenetics, used to study memory behaviour in animals (Thompson 1986; Liu et al. 2012; de Ortega‐San Luis and Ryan 2022). Although these methods rely on behaviour as a proxy for memory, our analysis deliberately sidesteps the deeper philosophical problem of what ‘reads’ any hypothetical engram to produce a cognitive experience. By isolating the search for the physical engram, we can more clearly assess the successes and challenges of a reductionist approach to memory.

Our central thesis is threefold. First, we elaborate on the philosophical toolkit within reductionist science. In doing so, we distinguish between reductionist methodologies and reductionist explanations. The former we defend as an indispensable strategy for generating causal knowledge, but the latter, we argue, is premature and likely insufficient for memory neuroscience. We evaluate the current state of engram research and highlight issues with different models. To make progress, we propose that the field adopt integrative pluralism—a philosophical framework for synthesising findings from multiple levels of investigation (Mitchell 2003). To facilitate this, we propose a reconceptualisation of the engram as a multiply realisable causal motif.

## 2 Reductionism

Reductionism may be regarded as either a powerful scientific method or a pejorative term for naive attempts to understand systems in terms of their simplest parts. Within philosophy of mind, the contention during the latter half of the 20th century was the reductionist thesis that mental processes can be reduced to neural processes or, more provocatively, fundamentally are brain processes (see Smart 1959, 1963). Put roughly, this view would hold that everyday memories—such as that you had porridge for breakfast this morning—are the outcome of nothing but biological mechanisms operating within assemblies of neural cells. Anti‐reductionists have variously challenged this form of reductionism on the basis that there is always something left over from the reduction—namely, subjective experience (Nagel 1974; Jackson 1982). Others have argued that psychological kinds—such as pain—are realised by multiple different structures and therefore cannot be identified with one structure alone (Putnam 1967). Neuroscientists proceed without having worked out a position on such debates, though it is presumed that studying biological mechanisms can tell us something about how various psychological kinds are produced. As for scientific inquiry itself, reductionism was conceived of as a programme for unifying the various sciences, thereby complying with intuitions that there is a final, fundamental science (Carnap and Black 1934). Today, the reductionist programme is more so driven by the intuition that actual causes exist at fundamental levels, indicating that causal explanations for phenomena will be achieved by investigating these lowest levels. This kind of reductionism—the reduction of scientific rather than mental phenomena—is the focus of this paper. To clarify what this means in practice for neuroscience, we first discuss the often‐conflated concept of ‘levels’ before turning to different kinds of reductionism.

### 2.1 Levelling With Reductionism

Reductionism is traditionally construed as implying or assuming levels. There are good reasons to be sceptical of the ‘levels’ concept as latching onto anything real or even providing a useful framework for scientists (see Potochnik 2017, 2021). That being said, the levels metaphor is undoubtedly intuitive to scientists and forms a ubiquitous undercurrent throughout the literature. Rather than defining terms like ‘levels’ precisely, we disambiguate three different ‘levels’ concepts to illustrate a conflation that occurs when making reductionist claims. These are levels of organisation, levels of analysis and levels of explanation.

**FIGURE 1:**
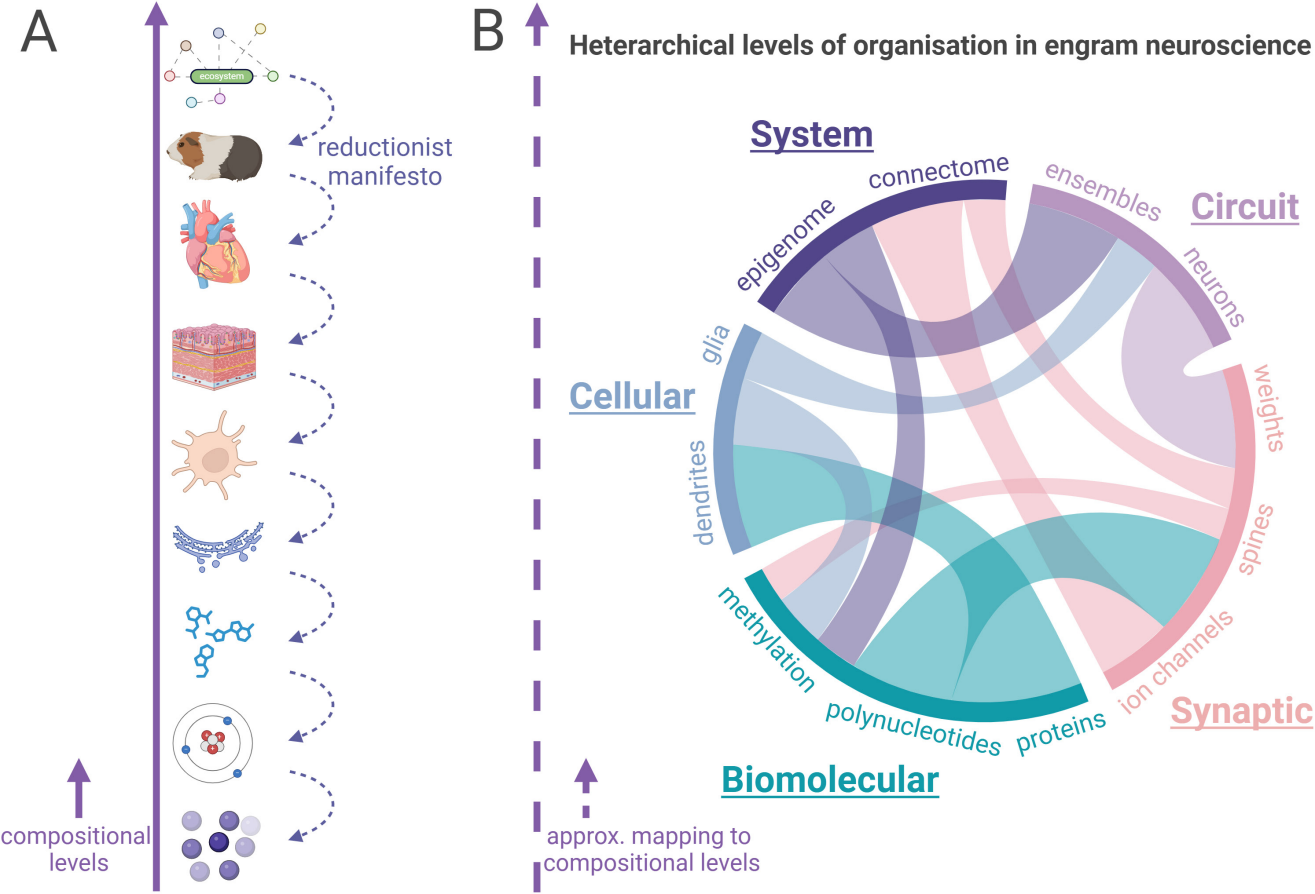
Levels of organisation as heterarchical. Levels of organisation are traditionally conceived as hierarchically organised compositions. Heterachies, in contrast, cross‐cut hierarchical structure in containing overlapping, mutually interacting entities that more accurately approximate the biological complexity that researchers face. As a note, we throughout the paper continue to refer to higher and lower levels of organisation that can be understood as referring to entities and processes at larger and smaller scales.

Levels of organisation are often conceived as a simple compositional hierarchy, where an organism is composed of organs, organs of tissues and so on, in a nested, part‐whole relationship (Figure 1A; but see Brooks (2021) for a more thorough treatment of the concept in science). From this follows the ‘reductionist hypothesis’, in that all animate or inanimate matter follows the same fundamental laws (Anderson 1972). These notions can be misleading and even erroneous in neuroscience, however. We suggest that organisational concepts like circuits, ensembles or networks are better understood as coarse‐grained models of a complex system, one in which overlapping and causally interacting structures cut across multiple compositional levels in a heterarchy (Figure 1B). For example, a single neuron can be a member of multiple organisational units (say, circuit X and ensemble Y) in the same way that a single drummer can play in both a rock band and a jazz quartet; both the band and quartet are real, distinct organisational units, yet neither is a ‘part’ of the other despite sharing components. Critically, this heterarchical view prohibits a hierarchical structuring in which a single, privileged ‘fundamental’ level exists. Although we depart from the intuitive notion of levels in advancing the heterarchical view, we think a heterarchy accords more with the organisation of various units in the brain.

**FIGURE 2:**
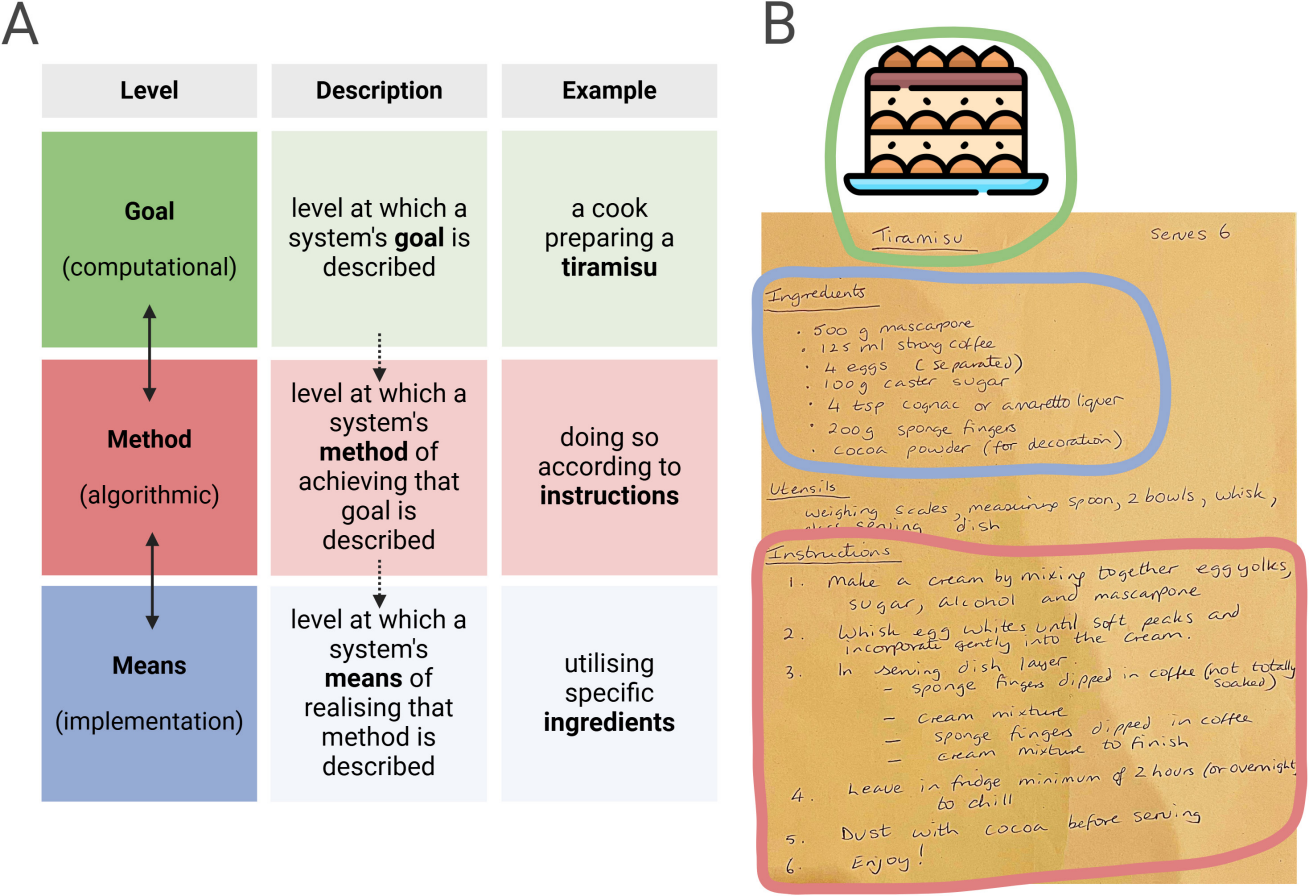
Levels of analysis. Levels of analysis demarcate the target system according to the particular perspective taken on the system, question asked of it or method used to study it. Marr's levels of analysis remain widely known and largely useful for neuroscience. These levels of analysis demarcate the target system according to the system's goals, its methods for achieving that goal and the means for which that method is implemented.

Next, levels of analysis are the different perspectives, questions and methods by which a phenomenon can be studied. The key behind levels of analysis frameworks is that understanding a phenomenon is not achieved by merely describing it nor knowing how to intervene to change it. The most familiar levels of analysis framework to neuroscientists will be David Marr's, which posits three levels of analysis by which a researcher can understand an information‐processing system—such as the visual system of the brain (Marr 2010; van der Helm 2014). The computational level is that at which the system's goal can be investigated; the algorithmic level is that at which the algorithm transforming input to output to meet that goal can be investigated; and the implementational level is the level at which researchers investigate the physical realisation of the algorithm. As a familiar example in Figure 2, cooks are well aware of the problem of preparing a dish (the goal/computational level) according to a recipe (the method/algorithmic level) that uses certain ingredients/utensils (the means/implementational level) (van der Helm 2014). Critical for any levels of analysis framework is that significant contributions can be made by studying a phenomenon at any single level and that all of these levels are epistemically equivalent (Lengyel 2024). Going forward, we will focus on the implementational level in our evaluation of engram neuroscience.

**FIGURE 3:**
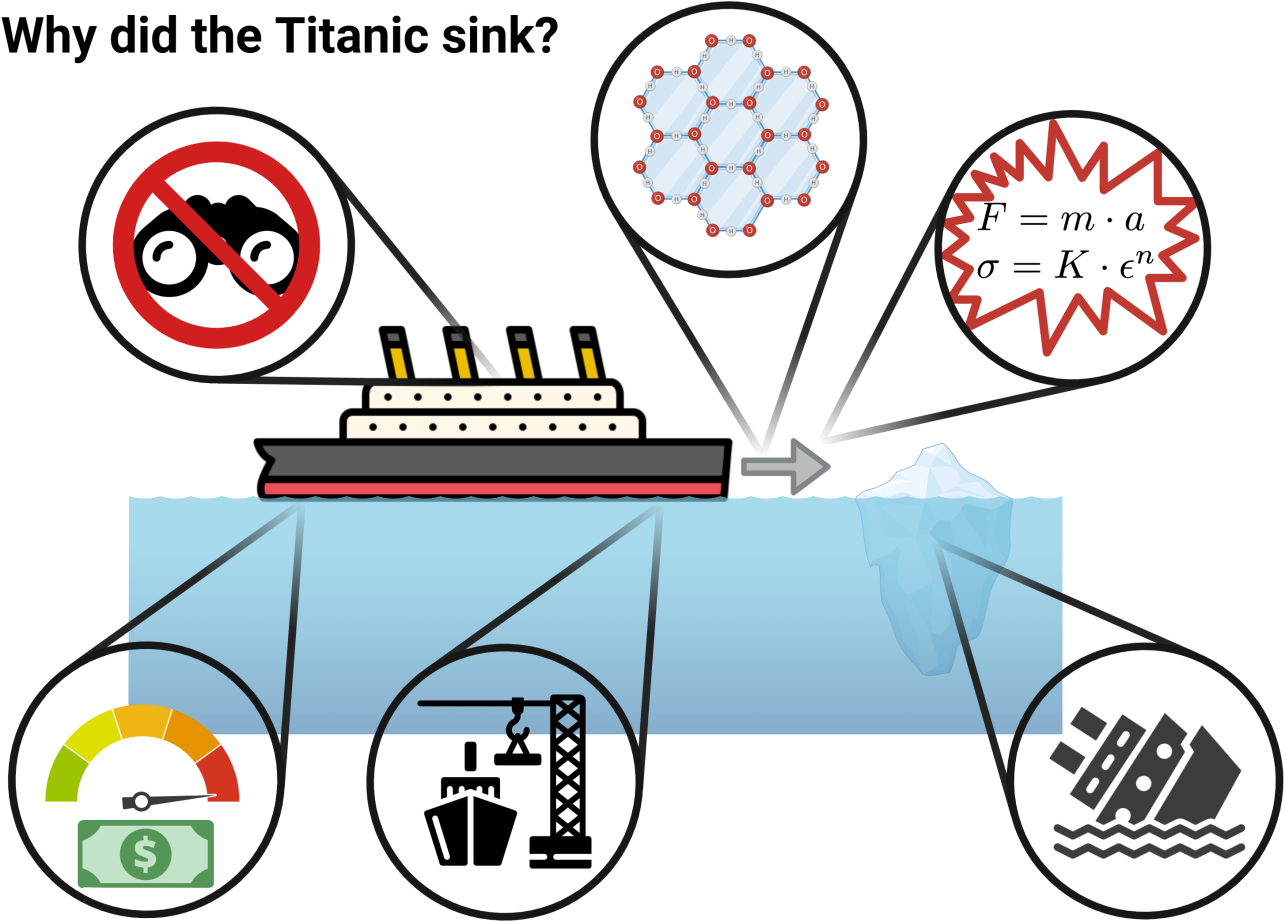
Levels of explanation. Levels of explanation illustrate the disparate phenomena that may be referred to in providing an explanation of some event, capacity or process. Levels of explanation may track levels of organisation (particularly heterarchical levels of organisation) or levels of analysis. The levels themselves are derived from particular perspectives taken on the system.

These levels should further be distinguished from levels of explanation. Levels of explanation are demarcated by the entities and details omitted or emphasised as important for explaining a given phenomenon (Barack and Krakauer 2021). Explanatory levels may track levels of organisation or levels of analysis, but they need not track one or the other and can involve multiple levels within each. In Figure 3, we see how there are various supposedly explanatory answers to the question of why the Titanic sank: An observer at the time may have said it was because the ship hit an iceberg; a physicist could purport that the force of the collision caused plastic deformation of the hull; an engineer could claim that low quality rivets and steel were used in construction; a historian could point out that the lack of binoculars caused the iceberg to be seen too late; a psychologist could suggest that the ship was going too fast, owing to the crew's pressure to complete the voyage as quickly as possible; and so on. As illustrated by this example, different explanatory levels are often derived from distinct perspectives on the relevant phenomenon. One goal of science is to identify the causal patterns that govern natural phenomena, allowing us to build predictive models that explain how the world operates. Achieving this goal can be done with reference to quite disparate phenomena at different levels of explanation. An explanation of memory in computational terms may involve individual neurons and mesoscale circuits, for example, or it may reference only energy landscapes in computational models without consideration of entities at various levels of organisation at all.

Although it may be tempting to claim that the implementational level is the fundamental level of organisation, this is erroneous: Such a reduction is orthogonal to Marr's framework, as any scale of organisation can be chosen as the implementational level. Thus, although Bickle (2015) is right that cellular and molecular methods have improved in their ability to test causal hypotheses, such causal evidence is insufficient for providing implementational, algorithmic, representational and computational evidence, which might be better sought at other organisational levels. We suggest that proponents of reductionism in neuroscience have conflated levels of organisation, levels of analysis and levels of explanation. That is, interventions occur at a specific level of organisation and offer a level of analysis that need not be a level of explanation. This point is important as we do not need to assume a priori that, for example, implementation must occur at the cellular level. Going further, we want to emphasise that it is not a priori determined which level of organisation or explanation should be privileged. This point will be important for evaluating reductionism.

### 2.2 Kinds of Reductionism

The general reductionist doctrine can be disambiguated as three theses: methodological, explanatory and metaphysical reductionism (Rosenberg 2020). We describe each in more detail below as being reductions of methods, explanations or things, respectively. Despite what might seem to be an alignment between these kinds of reductionism and the three levels concepts above, these forms of reductionism are distinct from the various levels concepts—the reductions we discuss here pertain to levels of organisation. Keeping the three kinds of levels and the three kinds of reduction distinct will bring needed clarity to our evaluation of neuroscientific memory research.

*Methodological reductionism* is the thesis that experimental interventions into parts and processes at lower organisational levels can provide valuable information for developing causal hypotheses and explanations of higher‐level phenomena. In practice, this means applying reductionistic heuristics—such as abstracting away variation, controlling contexts or simplifying inputs—as though reductionism is true, even though, for all we know, it could be false (Wimsatt 2006). A weak version of methodological reductionism is consistent with the idea that explanations require appeals to higher‐level structures and processes, such as those that are intermediary between cells and brain regions. One might argue, for example, that reductionist experiments, such as interventions into cells using optogenetics, can contribute to a theory that integrates data and evidence across levels. (This is the thesis we advance here.) A stronger version of methodological reductionism is the claim that lower‐level investigations provide evidence that will explain or correct higher‐scale ones. Minimally, proponents of methodological reductionism advocate for lower‐level investigations in order to substantiate existing higher‐level neurobiological, computational or psychological models and theories. We elaborate on such methodologies in Section 3. What is important for now is that methodological reductionism does not have to imply that reductionist methods of experimentation are capable of providing complete explanations.

Often, however, such methods are used in the service of achieving explanations. Here, we can understand explanations as causal models that show how a system's features or processes are brought about. Such explanations service understanding of the various dependency relations in the system and, in neuroscience, are often focused on discovering the circuits and mechanisms underlying various perceptual, cognitive and behavioural phenomena. *Explanatory reductionism* is the thesis that our exhaustive understanding of such phenomena is to be achieved by referring only to fundamental parts and processes at the lowest relevant levels. Such explanations involve omissions or simplifications of higher‐level phenomena, environmental factors and upstream and downstream processes—often because the relevant components were studied in isolation from the rest of the system (Kaiser 2011). In other words, proponents of explanatory reductionism think that a given behavioural phenomenon can be explained by appealing to lower‐level phenomena *alone*. In this way, there is a presupposition that there are no properties or features that solely appear in the aggregates of the fundamental parts or emerge at a higher level. However, the explanatory thesis need not deny their existence; the thesis merely states that such parts and processes are not going to play a significant, non‐redundant explanatory role in our theories.

A proponent of the methodological or explanatory theses need not be a *metaphysical* or *ontological reductionist*, who maintains that higher‐level parts and processes are fundamentally lower‐level parts and processes. This is not to say that higher‐level phenomena, such as systems and circuit phenomena, do not exist.^2^ Rather, the thesis is merely that higher‐level phenomena do not have any properties beyond those at lower levels. In other words, there are no properties or features that both emerge at higher levels and cannot be reduced to parts and lower‐level processes. The difference between the ontological thesis and the explanatory one is the difference between how the world really is and our mere understanding of it. Indeed, one can reject metaphysical reductionism, instead believing that there are non‐fundamental features or emergent properties of phenomena and yet defend the explanatory and methodological reductionist theses for pragmatic reasons. Altogether, the heart of reductionism is a challenge to the epistemic or ontological autonomy of higher‐level kinds from lower‐level, fundamental kinds. Making these distinctions between levels and kinds of reductionism explicit is crucial for the practising neuroscientist, as it helps clarify the precise claims being made by different research programmes, avoids inadvertently overstating the scope of experimental findings (e.g., assuming implementational findings automatically provide computational explanations), investigation and allows for a more nuanced evaluation of progress.

### 2.3 Limitations of Reductionism

Reductionism is not without its limitations. First, cellular and molecular mechanisms are overwhelmingly complex, and complexity can give rise to emergent properties not found in the lower‐level processes. Consider the formation of a flock of starlings that does not depend on any one bird's behaviour (Mitchell 2009) or a virtual governor regulating the average power demand of a system of generators (Hooker 1981). In both cases, there is a system, operation or body with properties not found in any of its component parts or processes. Along these lines, an anti‐reductionist might think that a population of neurons exhibits meaningful variance or conducts information processing over and above that of any individual neuron. For example, Ebitz and Hayden (2021) give a compelling account of neuronal populations as the fundamental computational unit in the brain. Of course, one can believe that some properties are emergent and yet also candidates for reduction (Wimsatt 2006). At the very least, work on populations of neurons, mesoscale circuits or gross anatomical structures might make cellular and molecular mechanisms more comprehensible than work appealing solely to lower levels. Tools that facilitate such abstraction and simplification (e.g., dimensionality reduction of neuronal population activity, electroencephalography [EEG] and functional magnetic resonance imaging [fMRI]) will provide a better framework as to how fundamental processes bear on higher‐level ones, and we welcome these advances.

Second, reductionist explanations involve a dismissal of potentially relevant higher scale factors or features of phenomena, such as system properties or features of the world external to the brain. Despite this, reductionist strategies often depend on theories and knowledge about higher‐level entities. For example, it was work on memory in experimental psychology and systems‐level research on hippocampal function that made neural interventions successful; higher‐level work provided reductionist neuroscientists with a target. Rather than challenging the autonomy of higher‐level systems and psychological explanations, such reductionist work might reinforce the necessity of these higher‐level kinds. Along similar lines, systems‐level research is a mesoscale intermediary that bridges multiple explanatory levels for a complete explanation of memory processes. Some argue for the priority of mesoscale models in understanding, in which case researchers begin at the mesoscale and then scale upwards and downwards (Batterman and Green 2020). At the very least, reductionist strategies cannot dismiss systems‐level and other higher‐level work. Reduction should be informed by good higher‐level theories.

Third, it could be argued that reductionism has yet to fulfil any promissory note of providing a complete explanation of behaviour, even in very simple and well‐known systems. This is commonly exemplified by the case of the nematode *Caenorhabditis elegans* : great advancements in systems and molecular biology have amounted to a comprehensive understanding of the composition of its nervous system (Cook et al. 2019; Taylor et al. 2021; Witvliet et al. 2021; Randi et al. 2023; Ripoll‐Sánchez et al. 2023), and yet, we still have a poor account of how this structure relates to its behaviour. Even in the case of a well‐studied circuit of ~30 neurons—the crustacean stomatogastric ganglion—issues of degeneracy have provoked serious challenges for reductionist methodologies to reveal how its limited functions emerge (Bargmann and Marder 2013; Marder et al. 2015). Evidently, attempting to infer ‘processes from processors’, or behaviour from biological structure, is an epistemological task of tremendous difficulty, even in a *simple* animal or circuit (Sternberg 2011; Krakauer et al. 2017). If we, as neuroscientists, are interested in understanding how the brain orchestrates behaviour, then looking solely at its lower‐level properties does not appear to be a profitable endeavour. Indeed, traversing epistemologically in this direction—inferring processes from processors—may even be impossible. As such, reductionist methodologies might be insufficient for explaining behaviour on their own, no matter how sophisticated or comprehensive the investigations may be.

Despite these limitations, a committed reductionist might argue that any perceived failure to explain behaviour is not a failure of the approach, but a reflection of the fact that we have only scratched the surface of the brain's fundamental mechanisms. New biological discoveries, the argument might go, will improve our understanding of these mechanisms. Persisting with our *C. elegans* example, it is known that the static anatomical details are insufficient. One putative key missing piece is a full, causal interactome—a dynamic map of all functional neuron–neuron interactions (Randi et al. 2023). It has been proposed that this could be used to attempt to reverse‐engineer the worm's nervous system in silico (Haspel et al. 2024), with success determined by a complete simulation of all brain states and behaviours. Although this may represent a neuroscientific ‘holy grail’ for reductionists (Sarma et al. 2018; Zhao et al. 2024), it is critical to note that describing or simulating all possible behaviours is not equivalent to providing an explanation for said behaviours. Mere descriptions do not provide insight into causal processes, and mere simulations only show how the system might work. As Haspel et al. note, true understanding requires using that hypothetical model to distil comprehensible principles and make testable predictions about the real animal. The road ahead is long, as even just producing such a simulation is a daunting task. Thus, the reductionist promissory note remains.

### 2.4 Benefits of Reductionism

Despite these limitations, there are reasons to think that a reductionist *methodology* can improve our understanding of neuroscientific phenomena, such as that of memory processes. Indeed, methodological reductionism has had many successes in the field. Consider that the study of the action potential by Hodgkin and Huxley (1952) or of long‐term potentiation (LTP) by Bliss and Lomo (1973) were achieved via reductionist methodologies (if not additionally securing an explanatory reduction, albeit of other neurobiological processes). We elaborate on the benefits of reductionism here.

Reductionist methodologies are a means to dissect causal processes into sub‐processes or components, thereby providing valuable insights into putative information‐processing and storage mechanisms that may implement memory phenomena. Some such causal processes almost certainly exist at lower levels, and identifying the functions of lower‐level components and processes can provide a means for correcting or otherwise improving our understanding of higher‐level components and processes. In this way, a reductionist methodology facilitates a more precise identification of these causal processes at lower levels. Even if it were the case that information‐processing and storage mechanisms exist solely in the aggregate or at higher levels of organisation, a continued search at lower levels can still advance understanding of the memory system—even if such understanding is solely that memory is realised at higher levels. We are optimistic about the prospects of uncovering aspects of information‐processing and ‐storage mechanisms at various levels of investigation.

Some have accepted methodological reductionism in the service of reductionist explanations. For example, Bickle (2016) argues that tool development is driven by the desire to test causal‐mechanistic hypotheses at lower levels of organisation, and successful intervention at such lower levels drives reductionist causal‐mechanistic explanations. Optogenetics, chemogenetics and molecular biology techniques are examples of reductionists' tools (Bickle 2020). According to Bickle, researchers use these reductionist tools to intervene into individual neurons to test causal‐mechanistic hypotheses about behaviour, thereby providing the basis for a reductionist explanation of cognitive phenomena. Such a reductionist methodology, for Bickle, is as successful as the causal evidence used to construct causal explanations. The successes of cellular and molecular intervention in the brain could therefore be thought of as evidence in support of the explanatory reductionist programme in neuroscience. However, we find this thesis too strong: Researchers may merely be gaining understanding of a brain phenomenon—of its structure, of features and properties or of correlational and causal relations among cells and molecules—without having explained cognition or behaviour (see Parker 2019).

One principal end towards which reductionist tools have been put in the last two decades is towards understanding the biological basis of learning and memory. Going forward, we discuss methodological reductionism as referring to research that employs reductionist tools (e.g., optogenetics, chemogenetics and a variety of other molecular biological techniques) and argue that they can offer valuable insights into the nature of memory. Moreover, we discuss explanatory reductionism as referring to reductionist theories and explanations for memory behaviour, arguing that such theories and explanations have so far been unsuccessful. Importantly, although methodological reductionism is in the service of explanations, these explanations need not themselves be reductionist: *It is possible to follow methodological reductionism without being an explanatory reductionist*. This point will be crucial for our appraisal of the search for engrams. Methodological reductionism can advance our understanding of memory systems by illuminating their causal features, whereas integration can improve our explanations of memory as well as account for properties that exist only at a higher level.

## 3 Reductionism and Memory Research

In separating explanatory reductionism from methodological reductionism, we can see how critiques levelled at explanatory reductionism (e.g., that current reductionist explanations are inadequate) do not in any way invalidate reductionist methodologies. This is an important assumption we make in our evaluation of the search for engrams in neuroscience. Philosophical and scientific enquiry about memory has traditionally been guided by the implicit assumption that some enduring physical change, or trace, in a system corresponds to storage of learned information (Robins 2017; De Brigard 2020; Najenson 2021). Such a trace would be causally linked to retrieval of the memory, in that the organism's capacity for recall is only possible owing to these physical changes.^3^ These ideas were formalised in the concept of an ‘engram’ by Richard Semon (1921): To Semon, the engram is biological changes that occur during learning and that can reoccur to cause memory behaviour are what constitutes an engram. Engram theory purports to explain how memories are stored within the nervous system and guides neuroscientific research on memory (Robins 2017). In this section, we provide an overview of competing mechanistic accounts (i.e., putative explanations) for where and how learned information, or memories, are stored within the brain. Critically, information storage is distinct from the related phenomena of encoding, consolidation, recall and forgetting (de Ortega‐San Luis and Ryan 2022). Much of the research on molecular mechanisms of memory conflates these distinct processes. Of course, a full reductionist explanation for memory would need to encompass these phenomena too. For now, however, we focus on memory storage.

### 3.1 The Synaptic Model

Neuroscience in the latter half of the 20th century has been guided by a hypothesis first formalised by Donald Hebb: that synapses are the principal site of information storage and that memories are formed via the associative modification of these synapses (Hebb 1949).^4^ In this view, the engram consists of a specific pattern of synaptic strengths within a neural network, as formed by the plasticity of these connection strengths (Martin et al. 2000). This model, which often aims for an explanatory reduction to the synapse, has been bolstered by a number of key experimental findings: alterations to synapses after memory formation in *Aplysia* (Castellucci et al. 1970; Pinsker et al. 1970); LTP and long‐term depression (LTD) of synaptic strength following specific patterns of neuronal activity (Bliss and Lomo 1973; Ito and Kano 1982); and fear conditioning in mice, resulting in LTP in auditory neurons of the amygdala (LeDoux 1995; Rogan et al. 1997). Each of these studies highlights that methodological reductionism can yield significant insights with interventions into cellular and molecular processes and measurements of memory via resulting behaviour (simply operationalised).

According to the model, long‐term changes are hypothesised to be governed by mechanisms like synaptic tagging and capture (STC) (Redondo and Morris 2011). The modern synaptic model is additionally focused not just on the plasticity of existing connections but also the growth or pruning of new ones. A particularly dynamic structure implicated in memory storage is dendritic spines (Kasai et al. 2010). Perhaps the most extensive model for memory storage comes from the *Drosophila* mushroom body. Connectomic and functional studies have shed light as to how sensory experience triggers specific molecular changes at identified synapses within a known circuit and how these alter the animal's future behaviour (Davis 2023). Memory engrams are posited to be stored in the specific synaptic connection between Kenyon cells and mushroom body output neurons (Aso and Rubin 2020), but the precise molecular and structural basis for the long‐term persistence of this synaptic modification is not yet understood.

Strong evidence causally links synaptic plasticity to learned behaviour, and the synaptic model has proven flexible in addressing challenges (Takeuchi et al. 2014; Langille and Brown 2018). Moreover, the model correctly distinguishes between the engram itself and the biomolecules that enable its formation; although ion channels, for example, are essential for inducing LTP/LTD, they are considered supportive machinery, not the stored information, at least on this account. Despite this, the model's strongest assertion—that synaptic weight changes are the primary and exclusive site of information storage—remains unproven. Testing these tenets is a key task for methodological reductionism, even if a complete explanatory reduction of memory to synaptic weights is unattainable at present.

### 3.2 The Molecular Model

Despite the dominance of the synaptic model, it has been challenged on a number of conceptual and empirical grounds (Gallistel and King 2009; Trettenbrein 2016; Abraham et al. 2019; Gershman 2023). An alternative model posits that the engram is stored as an intracellular molecular substrate, such as RNA or epigenetic marks (Gaito 1976; Gallistel 2021; Gold and Glanzman 2021; Gershman 2023). A key theoretical advantage of this molecular model is its proposal of a read–write mechanism, which can distinguish a transient role for synaptic plasticity in encoding from a more stable, molecular format for persistent storage (Langille and Gallistel 2020; Gallistel 2021). This pushes the search for an explanatory reduction of memory to a lower level of organisation than the synapse. Although theoretical frameworks for intracellular storage and readout exist (e.g., Akhlaghpour 2022; Mollon et al. 2023), substantial mechanistic validation remains a key challenge for the model as a whole (de Ortega‐San Luis and Ryan 2022).

Perhaps the most significant challenges to a purely synapse‐centric view come from studies of memory persistence. For instance, associative fear memories in rodents can be reinstated even after the specific synaptic connections strengthened during learning have been chemically erased or ‘depotentiated’ (Ryan et al. 2015). This suggests the underlying memory is stored independently from the change in synaptic weight (Tonegawa et al. 2015), a finding that is supported by evidence from a number of different organisms (de Ortega‐San Luis and Ryan 2022). That memory is stored independently of synaptic weights is bolstered by remarkable observations of memory surviving dramatic brain remodelling—such as insect metamorphosis or even entire head regeneration in flatworms—where the original synaptic architecture is completely broken down (Blackiston et al. 2015; Gershman 2023). These methodologically reductionist findings are difficult to reconcile within an explanatory framework focused solely on synapses, positioning the molecular model as the prominent candidate for how memory information persists.

Evidence in favour of the molecular model has come from reductionist science. For one, researchers have localised an associative memory trace within Purkinje neurons of the cerebellum (Johansson et al. 2014; Jirenhed et al. 2017), which is incompatible with the synaptic model. Additionally, a productive research programme for the molecular model has come from memory transfer experiments across individuals. What is powerful about these studies is the attempt to reduce an engram to a physical entity and then demonstrate a causal relation between that substrate and the behavioural expression of the memory. Horizontal transfer of certain forms of memories between organisms, proposed to be carried by RNA or RNA‐induced epigenetic mechanisms, has been demonstrated for invertebrates like *Aplysia* and *C. elegans* (Bédécarrats et al. 2018; Moore et al. 2021). Furthermore, vertical, or transgenerational, transfer of associative memories has been documented in organisms from worms and flies as well as possibly even rodents (Dias and Ressler 2014; Bozler et al. 2019; Moore et al. 2019; Kaletsky et al. 2020). It is important to note, however, that thus far transfer has only been demonstrated for simple memory phenomena. Another promising experimental avenue for the molecular model comes from memory phenomena in aneural organisms or cells, where the absence of synapses provides a sharp reductionist test case (Gershman et al. 2021; Gershman 2023; Kukushkin et al. 2024). Overall, evidence is mounting that molecular mechanisms play a fundamental role in memory storage separate from synaptic processes.

### 3.3 Supracellular Model

A tacit view in the neuroscientific literature holds that engrams have been discovered. These putative engrams are unique neuronal ensembles specific to the memory, the concomitant activity of which can be treated as a functional unit (Josselyn et al. 2015). Some findings indicate the substrate of a single memory is a distributed, brain‐wide engram complex, in which different components of a memory are suggested to be stored in ensembles across multiple brain regions (Josselyn and Tonegawa 2020). As such, the explanatory target for memory is shifted to a higher organisational level. Within this framework, one model for memory storage holds that a complete memory is encoded via structural plasticity of the wiring diagram, or connectome, of these ensembles (Chklovskii et al. 2004; Ryan et al. 2021). Such a model sidesteps issues of depotentiation that challenge the synaptic model (Ryan et al. 2015; de Ortega‐San Luis and Ryan 2022). However, some aspects of the supracellular model are complementary to the synaptic one. For example, dendrites have been proposed as fundamental units of the supracellular engram, capable of storing information through non‐linear operations (Kastellakis et al. 2023).

The causal role of these ensembles has been powerfully demonstrated by the targeted, methodologically reductionist tools of chemo‐ and optogenetics. Artificially reactivating neurons that were active during learning is sufficient to elicit the associated behaviour and can even be used to implant memories (Ramirez et al. 2013; Josselyn and Tonegawa 2020). This research programme is an exemplar of methodological reductionism, establishing a causal link between cell populations and behaviour at far greater specificity and control than Penfield's research using electrical probe stimulations (Penfield 1968; Josselyn et al. 2017). Furthermore, a recent study coupled chemogenetic tagging with electron microscopy and found that neurons involved in memory acquisition showcase experience‐dependent structural plasticity (Uytiepo et al. 2025). Although some supracellular models do propose alternative physical loci for the engram, the ensemble model has been the most successful. Despite these successes, findings from engram ensemble research do not yet outcompete the synaptic or molecular models for memory storage. Instead, they highlight a higher‐level framework that requires other models for a complete mechanistic explanation. Methodologically reductionist research will be essential for clarifying the precise nature of the engram.

Other limitations remain. The scope of proposed supracellular models for memory storage extends beyond neurons to include perineuronal nets (Tsien 2013) and glial cells (Caudle 2006; Kozachkov et al. 2025). Although there is increasing evidence that these structures can support neuron‐centric memory (Kol et al. 2020; Wang et al. 2020; Lev‐Ram et al. 2024; Williamson et al. 2024), direct causal evidence demonstrating non‐neuronal sites as primary storage loci for memories is lacking. Beyond these physical substrates, speculative hypotheses propose that engrams are electric field properties (Pinotsis and Miller 2023). Ephaptic coupling—the communication between neurons via local electric fields rather than direct synaptic or physical connections—is known to be important in the brain (Anastassiou et al. 2011; Han et al. 2020). However, it has proven notoriously difficult to study with a methodologically reductionist approach, as few tools can specifically manipulate a neuron's electric field to isolate its effects (Chen and Christopher 2020). Although currently lacking empirical support, these models of memory storage serve a valuable purpose: They challenge the assumptions of any reductive methodology focused solely on a view of the engram as a physical substrate to be identified and isolated. Undoubtedly there remains a great deal of biology out there awaiting discovery, and the conceptual space beyond current models of memory storage is vast.

### 3.4 Pluralistic Possibilities

Considerations of non‐material possibilities for memory storage raise an imperative point: There is no reason to assume that there is a single, universal substrate for the engram. Instead, memory could be multiply‐realisable, with evolution co‐opting a tremendous diversity of information‐storing mechanisms. This suggests that what neuroscientists label as ‘the engram’ may not be a single entity, but what Levin (2024) describes as a ‘heterarchical soup’ of competing and cooperating substrates. Although genetic information is constrained to a unified physical substrate (i.e., DNA) and single encoding structure (i.e., nucleotide bases) by reproduction, there is no equivalent evolutionary pressure demanding a single format for memory. Consequently, the physical basis of an engram could vary across species, individuals or even different memory systems within a single brain.

This view is supported by the biological principle of degeneracy, where structurally distinct elements can perform the same function (Albantakis et al. 2024). As argued by Kukushkin and Carew (2017), memory is not a monolithic process but a temporal hierarchy of mechanisms—from fast, transient kinase activity to slower gene expression and more stable structural or molecular changes. A memory, therefore, exists simultaneously at multiple timescales and in multiple mechanistic formats. Just as multiple gene networks can produce the same eye colour, it is plausible that a memory could be stored, say, by synaptic weights in one instance, an intracellular molecular state in another, or by the specific durational state of a molecular cascade—each serving as an engram at a different point in time.

This pluralistic model may, in fact, reflect universal principles of information storage that extend far beyond the brain. The conserved nature of memory phenomena in aneural organisms or cells is increasingly being recognised (Kukushkin 2018; Lyon et al. 2021; Gershman 2023; Kukushkin et al. 2024; Alberini 2025), and even inanimate systems like gene regulatory networks have associative memory (Biswas et al. 2021). This suggests that the fundamental ability to encode perturbations over time is not an invention of the nervous system but a deeply conserved property of life. As such, a reductionist search for *the* engram within the brain would be fundamentally misguided; the search would instead uncover a plethora of putative engrams as a subset of a universal biological toolkit for storing information, none of which exclusively constitute the basis of memory. This may reflect what we see in the field: The preceding sections highlight the vast number of findings uncovered by reductionist methodologies, and yet, any single explanatory reductionist framework is wanting. That there are many putative engrams is an important consideration for integrative attempts on competing models of memory storage and can serve as a hypothesis to be ruled out.

### 3.5 Current Competition and Future Resolution Between These Models

Each of these models (see Table 1) offers competing mechanistic explanations for memory behaviour. Colaço and Najenson argue that competing models cannot be integrated if they purport to explain the same phenomenon: In the case of the synaptic and molecular models, this shared explanandum is memory storage (Colaço and Najenson 2024). However, it is not clear whether these models must be incompatible because they involve different levels of organisation. As such, the success of optogenetic reactivation is not an automatic refutation of a synaptic or molecular model; rather, it highlights the need for more reductionist analysis to pinpoint the precise storage mechanism of memory, if such a thing exists. We show that the models might indeed be good candidates for integration.

**TABLE 1:** Memory models in neuroscience. Models of memory storage can be grouped into three families of models: synaptic, molecular and supracellular. Each kind of model postulates a unique locus of storage (first row) and draws supporting evidence from methods utilised at the target level of organisation, namely, synapses, molecules and ensembles (second row). The third row lists particular challenges that each kind of model faces or open questions that must be addressed. We also list particular relationships the model kind has with other model kinds in the fourth row. Models may be adversarial or complementary with each other.

	Synaptic model	Molecular model	Supracellular model
Primary locus of storage	The connection strengths (weights) between neurons in a network	Intracellular molecules (e.g., RNA, epigenetic marks, prions) within specific neurons	A specific, sparsely distributed ensemble of ‘engram cells’. Storage mechanism often left unspecified, but one proposal is the connectome of this ensemble
Key supporting evidence	LTP/LTD phenomena; correlation between synaptic changes and learning; STC hypothesis provides mechanism for specificity	Memory transfer experiments; persistence of memory through synaptic disruption; intracellular localisation of memory trace	Optogenetic/chemogenetic reactivation of cell ensembles is sufficient to elicit recall; brain‐wide mapping shows distributed nature of memory traces
Conceptual/empirical challenges	Synaptic instability; persistence of memory, despite depotentiation or synaptic breakdown; explaining memory transfer experiments	Lack of a clear ‘read‐out’ mechanism; explaining the specificity of memory recall; limited evidence in complex mammalian memory	What defines an ‘engram cell’? How is information specifically encoded in the network? How do memories persist despite connectomic breakdown? Is there memory in non‐neural cells?
Relationship to other models	Can be seen as competitive with the molecular model for the primary locus. Can be seen as the *implementation* of a supracellular model (i.e., synaptic weights define the ensemble)	Can be seen as competitive with the synaptic model, or as a complementary mechanism for long‐term stability (e.g., epigenetic changes ‘back up’ synaptic changes)	Can be seen as a higher‐level framework that is *realised* by synaptic and molecular mechanisms. Or the primary storage locus could be separate. The state of the ensemble (e.g., active vs. silent) may be regulated by molecular processes

One might argue that interventions at the higher level—for example, into networks of neurons—can be construed as gross interventions into lower‐level processes. In other words, ensemble reactivation via opto‐ or chemogenetics may actually be intervening on synaptic or molecular processes, thereby permitting recall of a stored memory. If this is the case, then it is possible that the successes of engram ensemble experiments are compatible with the synaptic or molecular models. Further reductionist analyses are evidently needed. Undoubtedly, synaptic, molecular and supracellular models of memory storage could theoretically coexist within the same explanatory framework, given that they involve distinct levels of organisation. Indeed, reductionist analysis has unveiled a vast array of molecular and cellular mechanisms that contribute to synaptic plasticity and long‐term memory traces (Kandel et al. 2014; Asok et al. 2019).

However, there are also competing, incompatible explanations for memory storage across models. In one compelling recent study, it was shown that canonical memory phenomena—the forgetting curve and massed‐spaced effect—are possible in non‐neural cells (Kukushkin et al. 2024). Many components of the molecular toolkit for memory formation were known to be conserved across cell types (Kukushkin et al. 2024), but critically, this study demonstrates that certain memory phenomena themselves can be reduced to biological units (i.e., cells) that lack synapses or neural circuitry. Moreover, although late phase LTP does involve at least some of the molecules proposed to mediate long‐term memory storage (Asok et al. 2019), the incompatibility between the synaptic and molecular model concerns what serves as an engram.^5^ Candidate molecular engrams—such as cytoplasmic macromolecules and epigenetic mechanisms in the nucleus—are often not those implicated in a synaptic model and are all theoretically capable of serving as engrams (Abraham et al. 2019; Akhlaghpour 2022; Gershman 2023). Thus, there is a tension between the models, because proponents of one memory storage model must find the resources to explain the others' experimental results. How do transfer experiments work if synapses are not transferred? Does the creation of potentiated synapses affect the candidate processes identified by molecular models? Research that attempts to resolve these disputes should prove fruitful for a satisfactory explanatory model in the future. Reductionist experiments in this vein will be critical for adjudicating between different models of where the engram is stored.

Some intriguing accounts have identified ways to unify some synaptic and molecular models together into a single theoretical framework. It has been argued, for example, that memory consists of *both* synaptic plasticity and concomitant intracellular biochemical changes (Langille and Brown 2018). Debates might persist as to what the *primary*, causal locus of the engram is in this model (is it synaptic changes activated by intracellular biochemical mechanisms, or instead intracellular biochemical changes expressed via synaptic plasticity?), but it is not clear that these mechanisms can be separated.

Others have proposed a dichotomy of memory storage functions, in which both synaptic and molecular models have a distinct role. Gold and Glanzman (2021) argue that synaptic plasticity represents temporary memory encoding, with select memories undergoing long‐term storage in the nucleus via epigenetic mechanisms. Gershman (2023) has alternatively proposed a unified framework where molecular and synaptic kinds of memory each serve different functions to solve an optimisation problem within the brain: An intracellular molecular mechanism stores ‘facts’ and the parameters of a generative model; computation is then facilitated by a complimentary inference model, which has its parameters stored at the synapse and is implemented by spiking activity in a network of neurons. It remains to be seen whether either of these accounts could then be integrated with a supracellular model (e.g., Ryan et al. 2021), where all three levels account for different aspects of memory processing, or whether these models are partially or entirely incompatible for more fundamental reasons (O'Sullivan and Ryan 2024). If supracellular entities indeed play a role in memory storage alongside synapses and molecules, then these models are no longer competing for *the* principal locus of the engram. Instead, the models represent complementary components of a multilevel system. Such unification attempts are made possible by showing that the models explain different phenomena; in particular, they dissociate explanatory levels of memory storage.

It will be interesting to experimentally assess whether these integrative theories hold any weight in vivo, and reductionist methodologies will be key for this. Until such accounts have been vindicated, however, it may prove fruitful to treat supracellular, synaptic and molecular models as competitors (Table 1). Doing so can drive the design of novel experiments, albeit the results must ultimately be synthesised within an integrative framework that can account for all available evidence. Alongside this, computational modelling represents a key tool in the integrative enterprise, via the potential to bridge different levels in a simulation of memory phenomena.

### 3.6 The State of the Field

In sum, the competing models of memory storage—molecular, synaptic and supracellular—have each spurred fruitful research programmes, yet no single model has provided a conclusive, all‐encompassing explanation for memory. Attempts are being made to resolve conflicts and build integrative frameworks which show great promise. The field has been invigorated by the advent of powerful methodologically reductionist tools that allow the precise labelling, tracking and manipulation of neuronal ensembles. The success of these methods is in providing evidence that reactivation of specific cell populations that were active during learning causes memory recall (Josselyn and Tonegawa 2020; de Ortega‐San Luis and Ryan 2022). Alongside this methodological triumph, as detailed in this section a vast array of methodologically reductionist studies have shed a great deal of light on the role of cellular and molecular processes in memory phenomena.

Methodologically reductionist tools are increasingly revealing a biological reality of profound complexity and dynamism that challenges the classical conception of the engram. At a cellular level, the notion of representational drift highlights that the specific neurons constituting a putative engram can change over time, even as the memory itself remains intact (Lopez et al. 2024; Tomé et al. 2024). Additionally, the discovery of silent engrams—physical traces that are inaccessible to natural retrieval cues but can be artificially reactivated—reveals that the engram can exist in different states of accessibility, complicating any simple causal link between physical substrate and behavioural recall (Josselyn and Tonegawa 2020). The increasingly recognised dynamism and flexibility of brain is thought to challenge the notion of the ‘stable engram’ (Zaki and Cai 2024). However, Robins (2020) provides good reason to think that stable engrams and neural dynamics are not incompatible: neural dynamics only implies that the implementation of memory storage need not be a simple one‐to‐one mapping between neural components and memory components. Understanding how memory storage is implemented is a major goal of memory neuroscience and a remaining challenge given neural dynamics and complexity.

This leaves the field in a paradoxical state: The very reductionist tools designed to find the engram at lower levels of organisation are revealing emergent, systems‐level properties that defy a simple reductionist explanation. Instead, we find a field that is data‐rich but understanding‐poor. In the next section, we address this challenge by providing a philosophical toolkit to advance understanding in engram neuroscience.

## 4 A Defence of Methodological Reductionism and Integrative Pluralism

In the last section, we showed that memory neuroscience has provided us with many possible, compatible models of memory storage that include biological phenomena at many levels of organisation. Given this state of affairs, we suggest that reductionist methodologies, although necessary for adjudicating among competing models, will be insufficient for engram discovery. Theoretical development is needed that is capable of integrating the rich, multilevel data the field is now generating. Such frameworks should define our explanandum, fix goalposts and set goals at each level of analysis. Importantly, it does not follow from this that there will be a right level of organisation to intervene into, nor does it settle in advance which level of explanation should be privileged, if any. Rather, the theoretical framework we offer provides an operationalisation of memory engrams that permits an integrative pluralism about neuroscientific memory research. In particular, we advocate pluralism about causal information‐processing and storage models and for their integration. Integration is a weaker notion than unification in that it permits the coexistence of multiple—perhaps even competing—models (Mitchell 2003), so we are not necessarily supporting any of the unification models described previously. We suggest that integrative pluralism necessitates a reconceptualisation of the engram and that this framework offers benefits to neuroscientists seeking explanations for memory phenomena. We end this section by addressing potential limitations of methodological reductionism for these purposes.

### 4.1 Reconceptualising the Engram

Viewing the synaptic, supracellular and molecular models as competing for the position of the principal locus of memory storage brings about fruitful theorising. One might press that competition may illuminate a means to adjudicate between models in an attempt to unify an explanatory framework. However, this operates on the assumption that there is a principal locus for the engram. Since Semon, the tacit view has been that the engram is a physical change, or collection of changes, in the brain that represent a memory. Much like how the gene was known long before its implementational and algorithmic details within the central dogma were unveiled, the assumption is there that there exists a physical manifestation for the engram and that finding it will reveal how the brain stores information. However, it is becoming evident that there exists a plurality of hypothetical information‐storing mechanisms in biology across organisational and evolutionary scales (Lyon et al. 2021; Kukushkin et al. 2024). As such, there exists a wealth of loci at which a researcher can intervene and thereby affect behaviour and memory. The issue is a lack of theorising about how such loci relate to behaviour and as such how to dissect and better understand the causal nexus of memory storage.

We propose that the current perspective, focusing on what the engram *is* as a physical entity, is misguided. Instead, we propose to define the engram in terms of what it *does*. As such, we support redefining the engram as a causal motif: a recurring pattern, which has causal influence by linking learning events to the reliable re‐enactment of a specific behaviour. Critically, a causal motif could be realised by different structures in different contexts, or indeed, these structures can operate together across contexts. This reconceptualisation clarifies a key point: the memory is not the structural change itself (say, the potentiated synapse, the phosphorylated molecule, the altered connectome); rather, the memory is the *process* of recall, which was made possible by a causal relationship established through these enduring physical changes. As attributed to Daniel Dennett, in a parallel debate over what the ‘right stuff’ for consciousness is, ‘it ain't the meat, it's the motion’ (Hofstadter 2007).

Consider a folded napkin. Under the prior framework, it could be argued that this holds a ‘memory trace’ of its folding, as defined by the set of stable changes that represent a memory, or store information. This indicates how wide and open the prior concept is: Any physical thing, even a folded napkin, can play the role of a memory trace or engram. In contrast, the engram‐as‐causal‐motif operationalisation takes the focus away from an inert trace (the meat) and instead emphasises its functional role (the motion). The causal efficacy that an inert trace has for re‐enacting a behaviour necessarily comes from the retrieval process. Additionally, this concept helps to clarify why neurons are special for memory. It is increasingly being recognised that memory phenomena are ubiquitous across the tree of life and that the origin of nervous system‐related genes and processes greatly predated the emergence of the nervous system (Kukushkin 2018; Lyon et al. 2021; Kukushkin et al. 2024). However, neurons are uniquely positioned to translate molecular and cellular adaptations into the systems‐level processes that manifest as behaviour and cognition, via the timescales of communication that they facilitate.

Operationalising engrams as causal motifs has a number of practical benefits to neuroscientists. First, it allows researchers to be precise about the causal variables involved in memory processes, which is necessary for identifying and individuating engrams (see Najenson 2025). Additionally, conceiving of engrams as causal motifs provides a promising theoretical upshot, as engrams are instead identified and defined as per their functional role. This is important, as engrams may be multiply realisable across diverse phyla, exist in various causal structures within individuals and play a role in experimental results that look very different across experimental contexts. Finally, causal motifs help to facilitate—or may even be a critical prerequisite for—integration into a unified framework. Conceiving of engrams as causal motifs allows diverse methodologies to be understood as probing different aspects or implementations of this common, multi‐scale causal structure. As such, this pluralism can be tamed with an integrative approach, guided by a clearer, functional definition of the engram. We believe that this will help to clarify the work that researchers are already doing and move the field towards explanatory frameworks for memory phenomena.

### 4.2 Defending Integrative Pluralism

Conceiving of engrams as causal motifs provides a straightforward means of integration of experimental outcomes via causal evidence. On Silva et al.'s (2014) account, interventions on some biological unit or process *A* can show whether *A* increases, decreases or has no effect on some downstream process or behaviour *B*. Such interventions are buttressed by measurements of both *A* and *B*, particularly for highly artificial interventions. (Such so‐called ‘connection experiments’ are further buttressed by Identity experiments and Tool experiments, which respectively contribute to identifying the relevant phenomenon and determining how to measure it. We focus on connection experiments as, with many putative engrams and many tools to identify them, getting precise about the relevant causal variables in these experiments and the causal roles they play will provide theoretical insight for further Identity and Tool experiments.) Drawing on this account, interventions into various putative engrams can illustrate the relation various units and processes have on memory behaviours. Integration across experimental outcomes will be strengthened by clarity about what precisely the putative engram is (e.g., synaptic weights and patterns of activity in populations of cells) and what precisely those units or processes effect, if anything. That is, integration via experimental outcomes allows researchers to get precise about characterising the engram and its role in memory processes. Interventions can provide understanding of interlevel relations as well. On Craver's (2002) account, interlevel experiments occur by intervening at one mechanistic level and measuring the result of that intervention at another mechanistic level. Such interlevel experiments identify lower‐level components and situate those components within a higher‐level mechanism, thereby providing many points of access to the mechanism. Experimental contexts in which neuroscientists intervene neurally and measure behaviourally (and vice versa) will involve interlevel experiments that are a means for integration in practice.

Pluralism is critical for integration. According to Gauld et al. (2022), there are four characteristics of integrative pluralism: (1) interdisciplinarity, (2) non‐exclusivity, (3) synchronicity of time intervals and (4) accumulation as the field develops. The first, second and third characteristics are achieved via pluralism: Multiple disciplines study memory, none has exclusive access to memory phenomena at some privileged level, and findings accumulate within and across disciplines. The third characteristic, that the models occur in some time interval, is achieved by integrating experimental outcomes via interventions and interlevel experiments. Integration across levels of analysis and organisation provides a means for higher‐level research to guide lower‐level research and for findings from reductionist methods to inform research at higher levels.

Integration is strengthened both through triangulation and via research programmes. Triangulation occurs when multiple methods are used to investigate a phenomenon (Kuorikoski and Marchionni 2016). The idea is that results from these methods converge to eliminate error and bias that come with any one method. Convergence comes in two forms (Roskies 2010). First, experimental results are interpreted in light of other empirical studies; individual results cannot be interpreted within the experimental context alone. These other empirical studies can be from the broader research domain—from psychology, neurology and physiology—or from other experiments using the same technique. Second, experiments that produce similar results using different task comparisons, in different laboratories, measuring the same subjects at different times or measuring different subjects all provide converging abductive evidence about some phenomenon. Such abductive evidence is available via reviews and meta‐analyses (Sullivan 2015). But triangulation is a stronger strategy when different methods are used in a way that validates the assumptions of any one method (Wright 2018). In this way, reductionist methods have integration built in; integration is achieved in practice through both interlevel experimental design and triangulation.

Research programmes also strengthen integration. Bickle et al. (2022) argue that research programmes sometimes use a divide‐and‐conquer strategy in which cellular and molecular processes are studied in multiple components and then integrated into a causal model of the system. Experiments are often designed for integration in this way (Bickle and Kostko 2018), but Bickle et al. further argue that integration has been facilitated by new methods that allow high‐resolution observations of circuits, thereby providing a levels‐less reduction of cognitive phenomena in which circuits are investigated *as* cells and molecules. In particular, miniscopes, iTango and Opto‐CCR5 have allowed researchers to investigate memory‐linking mechanisms in the molecules and cells of a cornu ammonis 1 (CA1) circuit. Of course, such an integration was not possible without higher‐level work on CA1 function, and this higher‐level work is being triangulated into the lower‐level investigation to determine what behavioural paradigm to use, which cells to target and what function to ascribe. Moreover, it is assumed that the relevant causal interactions only occur among cells and molecules and that these causal interactions are the target of inquiry. This assumption may seem unproblematic for fellow reductionists, but a healthy pluralism allows integration with population‐level computational work, systems‐level causal relations and findings from brain‐wide investigations as well as work in psychology and cognitive science to achieve a complete explanation of cognitive phenomena. As Darden (2016, 3) says it, ‘from an integrative perspective, reductive research shows its worth only when it makes contact with the higher‐level phenomena of interest’. Integrations with higher‐level work are sure to improve cellular and molecular models, just as research on memory linking in CA1 will be informative for higher‐level work.

These methods of integration show how investigations of phenomena integrate both levels of analysis and levels of organisation in unique ways. For example, researchers may intervene into mesoscale circuits to understand implementation or investigate the computational properties of individual neurons. Explanatory integration is complicated by this fact and, we argue, will be unsuccessful without a structuring theory. Engrams as causal motifs can serve this role until memory storage in the brain is better understood. Integration can also be facilitated by computational models that attempt to bridge levels, simulating how molecular or synaptic changes might give rise to ensemble properties or behavioural outcomes. Such a theory would provide a means for integration that services a better abduction about how brain processes give rise to cognitive and behavioural phenomena.

Pluralism can operate both at a community and individual level. But individualistic pluralism depends on the goals of each researcher. If one is interested in understanding a particular protein family, signalling mechanism or circuit for its own sake, there is still a place for this approach to science in integrative pluralism. We advocate such basic research. Nonetheless, if one is interested in how this knowledge can contribute to the development of neuroscientific theories, then awareness of how these discoveries might fit in the broader knowledge ecosystem of neuroscience, and the potential issues that arise in attempting to integrate them, is key.

Researchers aid in integrating their results across contexts, when doing so is warranted, by being explicit about the target of their intervention and how it relates to experimental results (especially behaviour). Being explicit showcases the aspect of the causal or functional structure that is targeted in a way that is apt for theorising and permits conceptual consistency across levels (Jacobs 2022). Achieving such conceptual consistency, facilitated by the careful distinctions between levels and types of reductionism discussed earlier, is paramount for successful integration. As for methodological reductionism, this bottom‐up strategy should make contact with top‐down strategies to help researchers on both ends determine which theories need revision. Indeed, understanding the relationships between models at various levels is required for integration (Burnston 2022), and understanding the relationship between models across various disciplines is also in the service of integration. This probe and revise strategy is a part of an epistemic iteration by which methods and explanations are progressively altered to facilitate model building and knowledge (Chang 2004). In this way, integration is an ongoing process (Brigandt, n.d.). That said, a theory of information storage, with the engram concept as a causal motif, will bring together and structure various research findings in the service of epistemic iteration.

### 4.3 Possible Objections

One worry about our proposal is that reductionist methodologies introduce distortions into the system by, for example, treating components in isolation (Kaiser 2011) and treating an open system as a closed one (Rose 1998). Simplification and abstraction are required for reductionist methodology to be fruitful, which will indeed distort the models that result (Chirimuuta 2024). Although distortions in models are inevitable—think of the old aphorism, ‘all models are wrong, some are useful’ (Box 1976)—here we think integration with other models can correct for some of these distortions. This will not be a complete solution, however. Parker (2022) argues that reductionist analyses are often successful for gaining insight, but achieving experimental tractability at lower levels requires ignoring properties relevant for explanatory purposes. We agree but remain optimistic that alternative models, integration and theoretical structuring can help to uncover these missing properties. We are more in spirit with Parker's (2021) claim that question‐driven experiments and conceptual advances can guide inquiry by providing a means to coherently integrate research at various levels.

One might still press then that, once we have a handle on all the relevant properties, we will have a reductionist explanation. In particular, the failure of reductionist explanations might just indicate that lower‐level models are currently wrong.^6^ The idea is that correct lower‐level models will reduce those higher‐level properties that currently seem emergent (see Barwich 2021). All the better for reductionist explanations, we say. But often these putative explanations are impoverished by explaining brain phenomena rather than behavioural phenomena. Although the former is in the service of the latter, we have yet to see a behavioural explanandum with causal explanans purely in terms of cells and molecules. We take the foregoing analysis to have shown this for memory.

Along similar lines, a committed reductionist might argue that the reason we lack a theory of cellular and molecular theory of behaviour—even in simple circuits or animals—is just that there is still much that remains unknown. More time and resources are needed to overcome existing methodological hurdles, and integration cannot remedy what is a lack of knowledge awaiting discovery. Although this is a possibility, it is yet another speculative note. And as we mentioned earlier, we remain sceptical that more detail alone will lead to new understanding. A risk we take in attempting to glean function from structure is that our hypotheses, the very theories we are testing, will be interpreted into the system (Rothman 2002). Integrative frameworks and theoretical constructs—such as the engram‐as‐causal‐motif concept—are needed to guide experimental design, help interpret findings across levels and prevent the post hoc imposition of hypotheses onto complex data.

Finally, one might still wonder *which* reductionist approaches are worthwhile. Surely not every reductionist method will be fruitful for informing higher‐level research, serving as a competitor or providing reductionist explanations. The answer to this question depends on one's purposes. And because there are many worthwhile reasons to model and understand memory at lower levels, it follows that there will be many kinds of reductionist methodologies that are suitable for fulfilling research aims. But insofar as models based on these approaches should be integrated with models in other areas of research, the right reductionist tools are those that help to dissect the causal nexus of the brain.

## 5 Conclusion

We started by disambiguating different kinds of levels and different kinds of reductionism, presenting these distinctions as a conceptual toolkit for practising neuroscientists. Doing so allowed us to focus on reductionist methods targeted at lower levels of organisation. These methods can be part of implementational, algorithmic or computational analyses, although we focused on implementational investigations here. The explanations that result from such investigations need not be reductionist explanations. Indeed, we find that there are many acceptable models in neuroscience at various levels, and we argued that more work should be done to integrate them and strengthen or falsify existing models. By assuming that reductionist methods provide reductionist explanations, researchers may inadvertently limit the scope of inquiry and conflate different kinds of levels and reductions in practice.

Our key point is this: Continue to be methodological reductionists. Keep doing opto‐/chemogenetics, transfer experiments, electrophysiology, connectomics and whatever method of choice. However, these methods alone will be insufficient for building complete models of memory storage. We need integration and theorising, both of which are currently under‐utilised strategies for getting a handle on memory in neuroscience. Critically embracing methodological reductionism, and distinguishing it from premature explanatory claims, is key for the permittance of active integrative pursuits. When this integrative pluralism is guided by clearer theoretical frameworks—such as reconceptualising the engrams as multilevel causal motifs—neuroscience can more effectively harness its powerful methodological tools to causally unravel the complexities of memory.

## Author Contributions

**Caitlin Mace:** conceptualisation. **Fionn O'Sullivan:** conceptualisation. **Scott R. Wilson:** conceptualisation.

## Funding

The authors have nothing to report.

## Conflicts of Interest

The authors declare no conflicts of interest.

## Data Availability

Data sharing is not applicable to this article as no datasets were generated or analysed during the current study.

## Endnotes

‘Mechanism’ is a technical term in philosophy of science. Here, we follow Ross and Bassett (2024, 84) in using ‘mechanism’ to refer to ‘a wide class of causal systems with different features’.

^2^
The thesis that only fundamental processes exist is called ‘eliminativism’, which we do not discuss here. Interested readers should see Churchland (1981).

^3^
This idea is defended against non‐engram views, such as Russell (1921), on the basis that the past cannot somehow reach forward into the present to exert influence over an organism's actions. Instead, there is a causal link between past experiences and an about‐to‐be‐performed behaviour by way of a dormant link in the causal chain that persists in the living changing organism until relevant.

^4^
The concept of the synapse as the site of information storage is integral to the adjacent field of artificial intelligence, in which learning and memory is enacted in neural networks via changes to their strengths or ‘weights’ (McCulloch and Pitts 1943; Rumelhart et al. 1986; Kohonen 1995). In this way, reductionist methodologies in neuroscience can be used to inform models in parallel or even completely peripheral fields.

^5^
We thank an anonymous referee for pressing for this clarification.

^6^
We thank an anonymous referee for this objection.
